# Temporal Association Among Influenza-Like Illness, Cardiovascular Events, and Vaccine Dose in Patients With High-Risk Cardiovascular Disease: Secondary Analysis of a Randomized Clinical Trial

**DOI:** 10.1001/jamanetworkopen.2023.31284

**Published:** 2023-09-14

**Authors:** Sheila M. Hegde, Brian L. Claggett, Jacob A. Udell, KyungMann Kim, Jacob Joseph, Michael E. Farkouh, Alexander Peikert, Ankeet S. Bhatt, Matthew C. Tattersall, Deepak L. Bhatt, Lawton S. Cooper, Scott D. Solomon, Orly Vardeny

**Affiliations:** 1 Brigham and Women’s Hospital, Harvard Medical School, Boston, Massachusetts; 2 Women’s College Hospital and Peter Munk Cardiac Centre, University Health Network, University of Toronto, Toronto, Ontario, Canada; 3 Department of Biostatistics and Medical Informatics, University of Wisconsin-Madison; 4 Brown University, The Warren Alpert Medical School, Providence, Rhode Island; 5 Kaiser Permanente Division of Research, Northern California, Oakland; 6 Department of Medicine, University of Wisconsin-Madison; 7 Mount Sinai Heart, Icahn School of Medicine at Mount Sinai, Mount Sinai Health System, New York, New York; 8 National Heart, Lung, and Blood Institute (NHLBI), National Institutes of Health (NIH), Bethesda, Maryland; 9 Department of Medicine, University of Minnesota, Minneapolis; 10 VA Minneapolis Health Care System, US Department of Veterans Affairs, Minneapolis, Minnesota

## Abstract

**Importance:**

Influenza-like illness (ILI) activity has been associated with increased risk of cardiopulmonary (CP) events during the influenza season. High-dose trivalent influenza vaccine was not superior to standard-dose quadrivalent vaccine for reducing these events in patients with high-risk cardiovascular (CV) disease in the Influenza Vaccine to Effectively Stop Cardio Thoracic Events and Decompensated Heart Failure (INVESTED) trial.

**Objective:**

To evaluate whether high-dose trivalent influenza vaccination is associated with benefit over standard-dose quadrivalent vaccination in reducing CP events during periods of high, local influenza activity.

**Design, Setting, and Participants:**

This study was a prespecified secondary analysis of INVESTED, a multicenter, double-blind, active comparator randomized clinical trial conducted over 3 consecutive influenza seasons from September 2016 to July 2019. Follow-up was completed in July 2019, and data were analyzed from September 21, 2016, to July 31, 2019. Weekly Centers for Disease Control and Prevention (CDC)–reported, state-level ILI activity was ascertained to assess the weekly odds of the primary outcome. The study population included 3094 patients with high-risk CV disease from participating centers in the US.

**Intervention:**

Participants were randomized to high-dose trivalent or standard-dose quadrivalent influenza vaccine and revaccinated for up to 3 seasons.

**Main Outcomes and Measures:**

The primary outcome was the time to composite of all-cause death or CP hospitalization within each season. Additional measures included weekly CDC-reported ILI activity data by state.

**Results:**

Among 3094 participants (mean [SD] age, 65 [12] years; 2309 male [75%]), we analyzed 129 285 person-weeks of enrollment, including 1396 composite primary outcome events (1278 CP hospitalization, 118 deaths). A 1% ILI increase in the prior week was associated with an increased risk in the primary outcome (odds ratio [OR], 1.14; 95% CI, 1.07-1.21; *P* < .001), CP hospitalization (OR, 1.13; 95% CI, 1.06-1.21; *P* < .001), and CV hospitalization (OR, 1.12; 95% CI, 1.04-1.19; *P* = .001), after adjusting for state, demographic characteristics, enrollment strata, and CV risk factors. Increased ILI activity was not associated with all-cause death (OR, 1.00; 95% CI, 0.88-1.13; *P* > .99). High-dose compared with standard-dose vaccine did not significantly reduce the primary outcome, even when the analysis was restricted to weeks of high ILI activity (OR, 0.88; 95% CI, 0.65-1.20; *P* = .43). Traditionally warmer months in the US were associated with lower CV risk independent of local ILI activity.

**Conclusions and Relevance:**

In this secondary analysis of a randomized clinical trial, ILI activity was temporally associated with increased CP events in patients with high-risk CV disease, and a higher influenza vaccine dose did not significantly reduce temporal CV risk. Other seasonal factors may play a role in the coincident high rates of ILI and CV events.

**Trial Registration:**

ClinicalTrials.gov Identifier: NCT02787044

## Key Points

**Question:**

Does high-dose compared with standard-dose influenza vaccine reduce cardiopulmonary events during periods of high, local influenza activity?

**Findings:**

In this secondary analysis of the INVESTED randomized clinical trial of 3094 participants from US sites with available weekly Centers for Disease Control and Prevention–reported influenza-like illness (ILI) activity, ILI was temporally associated with cardiopulmonary and cardiovascular (CV) hospitalizations. However, high-dose compared with standard-dose influenza vaccine did not significantly reduce occurrence of the primary outcome.

**Meaning:**

The findings indicate that influenza activity was temporally associated with CV events in patients with high-risk CV disease, and temporal CV risk was not significantly reduced by a higher influenza vaccine dose.

## Teaser

This secondary analysis of the INVESTED randomized clinical trial assesses whether high-dose compared with standard-dose influenza vaccine was temporally associated with cardiovascular (CV) events among patients with high-risk CV disease during periods of high influenza-like illness activity.

## Introduction

Influenza has been associated with increased risk of cardiopulmonary (CP) events, including myocardial infarction (MI) and heart failure (HF).^1,2,3,4,5,6,7,8^ Proposed mechanisms that suggest an association between influenza infection and cardiovascular (CV) risk include induction of systemic effects via immune stimulation and inflammation that can provoke plaque rupture, increased metabolic demand, adrenergic surge, hypoxia, hypercoagulability, and direct myocardial toxic effects.^9,10^ Seasonal outbreaks of influenza occur primarily in winter months when transmission may be more favorable, and CV events demonstrate a similar temporal pattern.^11^ Time-series analyses have also demonstrated an association between influenza and CV events in observational and retrospective studies.^12,13^ Influenza vaccine may be involved in reducing adverse CV outcomes, as suggested by observational and randomized clinical trials.^14,15,16,17,18,19^

The Influenza Vaccine to Effectively Stop Cardio Thoracic Events and Decompensated Heart Failure (INVESTED) trial assessed the efficacy of high-dose trivalent or standard-dose quadrivalent influenza vaccine in North American participants with high-risk CV disease during the 2016 to 2019 influenza seasons.^20^ The primary end point was time to first CP hospitalization or all-cause death in each season. High-dose vaccine was not superior to standard-dose vaccine for the primary end point, which assessed benefit from 2 weeks following vaccination through the end of July of each influenza season.^21^ Whether treatment efficacy may be different during periods of high influenza-like illness (ILI) activity is not known. In this analysis, we compared locally defined ILI activity, as provided by the Centers for Disease Control and Prevention (CDC), with outcomes in INVESTED and assessed whether high-dose compared with standard-dose influenza vaccine modified the events in association with locally determined ILI activity.

## Methods

**Figure 1.**
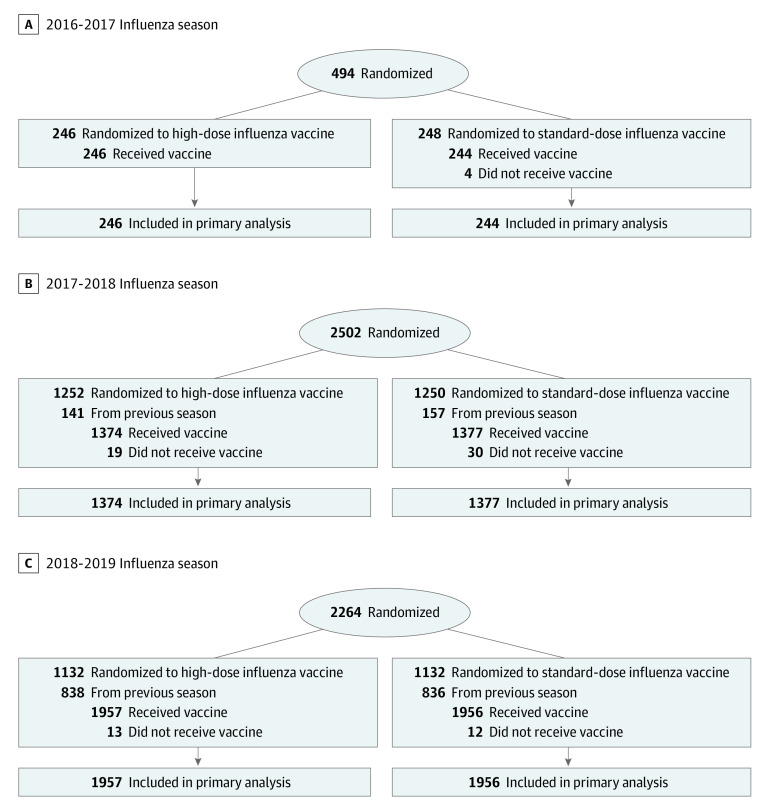
CONSORT Diagram The number of patients screened was not collected.

This secondary analysis of a randomized clinical trial was a prespecified analysis of the INVESTED trial, a pragmatic, randomized, double-blind, active comparator trial conducted at 157 participating centers in the US and Canada from September 2016 to July 2019. Follow-up was completed in July 2019. Patients with high-risk CV disease previously hospitalized for either MI in the past 12 months or HF in the past 24 months were randomized to high-dose or standard-dose influenza vaccine. The protocol and primary results have been previously published (Supplement 1 and Figure 1).^21^ This study was a preplanned analysis of the parent trial and follows the Consolidated Standards of Reporting Trials (CONSORT) reporting guideline for randomized studies.^22^ Ethics committees at each enrolling site approved the protocol or the review was ceded to a central institutional review board. All participants provided written informed consent in accordance with established guidelines.

### Association Between ILI and CV Events

Influenza-like illness was defined based on the percentage of visits to sentinel clinicians for fever (temperature ≥37.8 °C) and a cough and/or a sore throat without a known cause other than influenza as reported to the CDC US Outpatient Influenza-Like Illness Surveillance Network. Therefore, ILI could have included any respiratory pathogen presenting with these symptoms. We included CDC data from 108 US sites and territories; sites from Florida and Canada were not included due to unavailable weekly ILI data. Race categories included Asian, Black, First Nations or American Indian, White, and other (Native Hawaiian, Pacific Islander, >1 race, chooses not to report, does not know, and not available or missing), and ethnicity categories included Hispanic or Latino, non-Hispanic or non-Latino, and other (chooses not to report, does not know, and not available or missing). Race and ethnicity were ascertained by self-report.

### Statistical Analysis

#### Primary and Secondary Outcomes

Data were analyzed from September 21, 2016, to July 31, 2019. We compared publicly available, weekly, CDC-reported, state-level ILI with the weekly occurrence of the primary outcome (composite of all-cause death or CP hospitalization) and secondary outcomes (death, CV hospitalization, and pulmonary hospitalization) among US participants from September 2016 to July 2019 using logistic regression models. Weekly ILI activity from 0 to 9 weeks prior to (lag 0 to lag 9) the outcome of interest was considered to assess the temporal association between exposure and outcome. Model 1 was adjusted for state, demographics (age, self-reported sex, and race) and enrollment strata (history of MI, history of hospitalization for HF). Model 2 was additionally adjusted for CV risk factors (diabetes, body mass index >30 [calculated as weight in kilograms divided by height in meters squared], kidney impairment, current smoker, peripheral arterial disease, ischemic stroke, hypertension, hyperlipidemia, asthma, chronic obstructive pulmonary disease, percutaneous coronary intervention, coronary artery bypass graft, atrial fibrillation, and implantable cardioverter-defibrillator).

#### Treatment Outcome

To assess the treatment outcome, we used logistic regression models to evaluate the weekly odds of the primary outcome for the high-dose compared with standard-dose vaccine treatment group adjusting for state to account for possible regional differences. In a sensitivity analysis, we analyzed the association between ILI activity and outcomes (1) only during months with typically higher influenza activity (September 15th to May 15th of each enrolling season) and (2) during weeks with higher ILI activity (>2 SDs above a state’s mean ILI activity). Last, we compared weekly ILI with outcomes adjusting for state, vaccine treatment assignment, and month, referenced to the month of July.

Analyses were performed using Stata, version 14 (StataCorp LLC), and statistical significance was set at 2-sided *P* < .05. *P* values were not adjusted for multiple testing.

## Results

**Figure 2.**
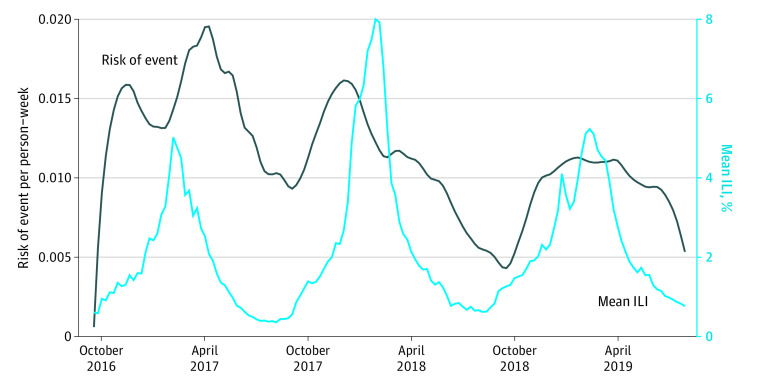
Temporal Risk of the Primary Outcome Association between risk of the primary outcome (composite of cardiopulmonary hospitalization or death) per patient-week and mean influenza-like illness (ILI) activity over time.

In this sample of 3094 US participants (mean [SD] age, 65 [12] years; 781 [25.2%] were female, 2309 [74.6%] were male, and 4 participants did not report sex [0.1%]). Of these, 30 (1.0%) were Asian, 690 (22.3%) Black, 12 (0.4%) First Nations or American Indian, 226 (7.3%) Hispanic or Latino, 2832 (91.5%) non-Hispanic or non-Latino, 2238 (72.3%) White, 124 (4.0%) other race, and 36 (1.2%) other ethnicity. Baseline characteristics and concomitant therapies were well balanced between treatment groups (Table 1). The majority of enrolled participants (2352 [76.0%]) had an HF qualifying event, 672 (21.7%) had prior HF hospitalization, and 375 (12.1%) had a prior MI event. The sample associated 129 285 person-weeks of enrollment with 1396 composite primary outcome events (1278 CP hospitalizations, 118 deaths), 322 deaths, 1141 CV hospitalizations, and 183 pulmonary hospitalizations. A lag of 1 week was chosen for the logistic regression analyses relating ILI activity to the primary and secondary outcomes via forward stepwise selection models (eTable in Supplement 2). Mean ILI activity over time, as reported by the CDC and risk of the primary outcome over time are shown in Figure 2. Mean ILI activity was the highest in the 2017 to 2018 season.

**Table 1.** Baseline Characteristics of Participants in the Sample Compared With Participants in the INVESTED Trial

Characteristic	Sample influenza vaccine, No. (%)		INVESTED trial, No. (%) (n = 5260)
	High dose (n = 1548)	Standard dose (n = 1546)	
Randomization y			
2016-2017	161 (10.4)	162 (10.5)	494 (9.4)
2017-2018	761 (49.2)	769 (49.7)	2502 (47.6)
2018-2019	626 (40.4)	615 (39.8)	2264 (43.0)
Age, mean (SD), y	65 (13)	67 (13)	66 (13)
Sex			
Female	395 (25.5)	386 (25.0)	1473 (28.0)
Male	1152 (74.4)	1157 (74.8)	3773 (71.7)
Race			
Asian	12 (0.8)	18 (1.2)	155 (2.9)
Black	336 (21.7)	354 (22.9)	784 (14.9)
First Nations or American Indian	6 (0.4)	6 (0.4)	49 (0.9)
White	1130 (73.0)	1108 (71.8)	4103 (78.0)
Other^a^	64 (4.1)	60 (3.9)	169 (3.2)
Ethnicity			
Hispanic or Latino	121 (7.8)	105 (6.8)	517 (9.8)
Non-Hispanic or non-Latino	1410 (91.1)	1422 (92.0)	4683 (89.0)
Other^b^	17 (1.1)	19 (1.2)	60 (1.1)
Ejection fraction, mean (SD), %	41 (17)	42 (16)	42 (16)
BMI, mean (SD)^c^	31.9 (8.1)	31.7 (7.7)	30.9 (7.4)
Qualifying event			
HF	1170 (75.6)	1182 (76.5)	3289 (62.7)
MI	377 (24.4)	361 (23.4)	1960 (37.3)
Eligibility risk factor			
Age >65 y	854 (55.2)	862 (55.9)	2987 (56.9)
Current BMI >30^c^	823 (53.2)	833 (54.0)	2551 (48.6)
Current or past LVEF <40%	758 (49.0)	751 (48.7)	2208 (42.1)
Type 1 or type 2 diabetes	610 (39.4)	630 (40.8)	1950 (37.2)
History of chronic kidney disease	567 (36.7)	573 (37.1)	1587 (30.2)
Current tobacco smoker	219 (14.2)	261 (16.9)	902 (17.2)
Prior HF hospitalization	341 (22.0)	331 (21.5)	903 (17.2)
Prior MI	187 (12.1)	188 (12.2)	745 (14.2)
History of ischemic stroke	138 (8.9)	132 (8.6)	433 (8.3)
History of peripheral artery disease	79 (5.1)	80 (5.2)	232 (4.4)
Other medical or surgical history			
Hypertension	1250 (80.8)	1211 (78.5)	4046 (77.1)
Dyslipidemia	1066 (68.9)	1058 (68.6)	3616 (68.9)
Percutaneous coronary intervention	524 (33.9)	529 (34.3)	2162 (41.2)
Atrial fibrillation	591 (38.2)	597 (38.7)	1725 (32.9)
Coronary artery bypass graft	308 (19.9)	317 (20.5)	1040 (19.8)
Chronic obstructive pulmonary disease	340 (22.0)	338 (21.9)	1006 (19.2)
Implantable cardioverter-defibrillator	371 (24.0)	368 (23.8)	956 (18.2)
Asthma	175 (11.3)	192 (12.4)	602 (11.5)
Canadian Cardiovascular Society grading classification^d^			
I	179 (54.6)	186 (57.6)	1116 (61.2)
II	98 (29.9)	86 (26.6)	498 (27.3)
III	39 (11.9)	44 (13.6)	142 (7.8)
IV	12 (3.7)	7 (2.2)	68 (3.7)
New York Heart Association Functional Classification^d^			
I	176 (15.8)	177 (15.8)	543 (17.2)
II	546 (48.9)	529 (47.1)	1584 (50.1)
III	354 (31.7)	377 (33.6)	931 (29.4)
IV	41 (3.7)	40 (3.6)	104 (3.3)
Maintenance medications for those with MI as index event			
Statins	346 (91.8)	329 (91.1)	1785 (91.1)
Aspirin	335 (88.9)	326 (90.3)	1835 (93.6)
β–Blocker	342 (9.7)	316 (87.5)	1679 (85.7)
Maintenance medications for those with HF as index event			
β–Blocker	988 (84.4)	990 (83.8)	2779 (84.5)
Diuretic	970 (82.9)	975 (82.5)	2607 (79.3)
ACE inhibitor, ARB, or ARN inhibitor	765 (65.4)	780 (66.0)	2197 (66.8)
Mineralocorticoid receptor antagonist	391 (33.4)	410 (34.7)	1118 (34.0)
Digoxin	113 (9.7)	101 (8.5)	314 (9.6)

Abbreviations: ACE, angiotensin-converting enzyme; ARB, angiotensin receptor blocker; ARN, angiotensin receptor neprilysin; BMI, body mass index; HF, heart failure; INVESTED, Influenza Vaccine to Effectively Stop Cardio Thoracic Events and Decompensated Heart Failure; LVEF, left ventricular ejection fraction; MI, myocardial infarction.

^a^ Other race included Native Hawaiian, Pacific Islander, more than 1 race, chooses not to report, does not know, and not available or missing.

^b^ Other ethnicity included chooses to report, does not know, and not available or missing.

^c^ Calculated as weight in kilograms divided by height in meters squared.

^d^ Limitation classifications: I, none; II, slight; III, moderate; and IV, severe.

### Association Between ILI Activity and CV Events

A 1% ILI increase in the prior week was associated with an increased risk in the primary outcome of composite of CP hospitalization or all-cause death (odds ratio [OR], 1.14; 95% CI, 1.07-1.21; *P* < .001), CP hospitalization (OR, 1.13; 95% CI, 1.06-1.21; *P* < .001), and CV hospitalization (OR, 1.12; 95% CI, 1.04-1.19; *P* = .001) after adjusting for state, demographic characteristics, enrollment strata, and CV risk factors (Table 2). No association was found between increased ILI activity and pulmonary hospitalizations (OR, 1.18; 95% CI, 0.99-1.40; *P* = .06). Increased ILI activity was not associated with all-cause death (OR, 1.00; 95% CI, 0.88-1.13; *P* > .99).

**Table 2.** Association of Weekly Odds of Primary and Secondary Outcomes With Influenza-Like Illness Activity^a^

	Events, No.	Model 1, OR (95% CI)^b^	*P* value	Model 2, OR (95% CI)^c^	*P* value
Composite primary outcome	1396	1.14 (1.07-1.22)	<.001	1.14 (1.07-1.21)	<.001
Cardiopulmonary hospitalization	1278	1.14 (1.07-1.21)	<.001	1.13 (1.06-1.21)	<.001
All-cause death	322	1.00 (0.89-1.14)	>.99	1.00 (0.88-1.13)	>.99
CV hospitalization	1141	1.12 (1.05-1.20)	.001	1.12 (1.04-1.19)	.001
Pulmonary hospitalization	183	1.18 (0.99-1.39)	.06	1.18 (0.99-1.40)	.06

Abbreviations: CV, cardiovascular; OR, odds ratio.

^a^ Patient-weeks of influenza-like illness activity were associated with outcomes with a lag of 1 week.

^b^ Model 1: adjusted for state, age, sex, race, history of myocardial infarction, and history of heart failure.

^c^ Model 2: adjusted for model 1 and diabetes, body mass index >30 (calculated as weight in kilograms divided by height in meters squared), kidney impairment, current smoker, peripheral arterial disease, ischemic stroke, hypertension, hyperlipidemia, asthma, chronic obstructive pulmonary disease, percutaneous coronary intervention, coronary artery bypass graft, atrial fibrillation, and implantable cardioverter-defibrillator.

**Figure 3.**
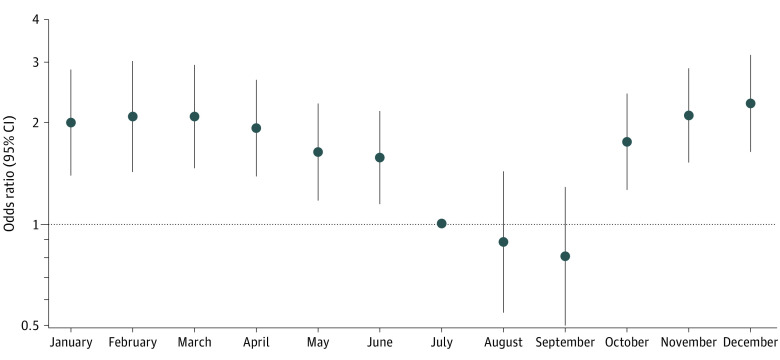
Temporal Risk of the Primary Outcome by Month Independent of Influenza-Like Illness Activity Weekly odds of the primary outcome (composite of cardiopulmonary hospitalization or death) by month (referenced to July), after adjusting for influenza-like illness, treatment, and state.

Warmer months (July, August, and September) were associated with a lower risk of events even after adjusting for ILI (Figure 3). Figure 3 demonstrates the weekly odds of the primary outcome by month (referenced to July) after adjusting for ILI, vaccine treatment assignment, and state.

### Treatment Effect

Evaluation of the weekly odds of the primary outcome by treatment yielded similar results to the primary results from the INVESTED trial. High-dose was not superior to standard-dose vaccine in its association with risk of the primary outcome (CP hospitalization or death) in the prespecified primary analysis after adjusting for state (OR, 1.07; 95% CI, 0.95-1.20; *P* = .25), when restricting analysis to months with typically higher influenza activity (OR, 1.09; 95% CI, 0.97-1.24; *P* = .15), or during weeks of high ILI activity (OR, 0.88; 95% CI, 0.65-1.20; *P* = .43) in this temporal analysis.

## Discussion

In this secondary analysis of the INVESTED randomized clinical trial, ILI was temporally associated with CP and CV hospitalization but not with all-cause death in patients with high-risk CV disease. A higher dose of trivalent influenza vaccine compared with standard-dose quadrivalent influenza vaccine did not modify this association, even after restricting the analysis to months with typically higher influenza activity or weeks in which ILI activity was high. Warmer months in the US were associated with lower CV risk independent of local ILI activity.

The primary end point of INVESTED was designed to capture CP events that occurred during the broad influenza season, but not necessarily during times of high influenza activity, because we hypothesized that if influenza infection led to CV and pulmonary events, the events might occur even several months after a primary infection. Nevertheless, we anticipated the possibility that modification of these events might be more temporally associated with influenza infection. This prespecified analysis was designed to assess whether high-dose influenza vaccine would provide greater benefit than standard-dose vaccine during times of high influenza activity. That we did not observe a benefit in this analysis suggests that in a high-risk population, the higher dose of vaccine was not associated with modification of events that were extremely likely. However, the benefit of any influenza vaccine compared with placebo in this population cannot be determined from this analysis. Based on other data comparing vaccination with placebo, it is likely that influenza vaccination with either dose would have more substantial benefits over placebo in patients with high risk.^23^ Moreover, as the influenza virus is not the only circulating virus during the winter months, it is possible that other respiratory viruses may contribute to the association between time and CV events.

In patients with high-risk CV disease, ILI activity was temporally associated with CP and CV hospitalizations. Although a similar OR for pulmonary-related hospitalizations was present, there was no association with increased ILI activity, which may in part be attributed to the small number of events (n = 183) and underscores the relatively higher occurrence of CV (n = 1141) compared with pulmonary events in this sample. Furthermore, individual-level ILI or infection was not assessed in this sample, so the true incidence of influenza is not known. Nevertheless, the temporal association with CV events supported prior findings of emergency department visits in New York City of the association of ILI with CV mortality and in observational data in an older cohort of the association of ILI with MI and HF events.^12,13^ This association remained when the analysis focused on months with typically higher influenza activity and weeks with high ILI activity.

Although all participants received influenza vaccine, the temporal association between ILI activity and CV events remained. Additional analysis demonstrated that warmer months were associated with lower risk of CV events after accounting for ILI activity and vaccine dose, suggesting that other seasonal factors may also play a role in the seasonal variation in CV events. Vaccine effectiveness was not associated with a modification in the seasonality of ILI and CV events in another analysis of New York state data.^24^ Unmeasured environmental factors, such as temperature, absolute and relative humidity, and wind speed, have been associated with influenza outbreaks and CV events with some variation by influenza subtypes and may explain some of the seasonal variation.^25,26,27,28,29^ Registry data from Sweden have similarly demonstrated the impact of seasonal environmental factors, which attenuated the association between higher incidence of MI and higher incidence of influenza.^30^

### Limitations

Several limitations of this analysis should be noted. Inferences about the association between patient-level influenza illness or other respiratory viruses and subsequent events could not be made, as the respiratory influenza infection status of participants was not assessed in this study. State-level ILI activity may not have represented regional-level or patient-level ILI activity. Only states with available ILI activity were included in this analysis, which limited generalizability to participants, excluding those in Florida and Canada. That we did not randomize patients to placebo prevented us from drawing conclusions about the impact of any influenza vaccination on the temporal association between ILI activity and CV events. Also, the difference in valence between the high-dose trivalent influenza vaccine and the standard-dose quadrivalent vaccine may have contributed to the absence of outcome modification of the vaccine dose, with the standard-dose vaccine providing additional coverage for the influenza B/Yamagata strain. Changes in seasonal temperature, humidity, weather patterns, and other environmental factors were not accounted for in this analysis. Additionally, the trial had limited power for this prespecified secondary analysis.

## Conclusions

In this secondary analysis of the INVESTED randomized clinical trial evaluating the efficacy of high-dose trivalent vaccine compared with standard-dose quadrivalent vaccine in reducing CP events among patients with high-risk CV disease in the US, influenza activity was temporally associated with an increasing risk of CP events, yet a higher-dose influenza vaccine did not significantly reduce temporal CV risk. Other seasonal factors may also play a role in the higher rate of CV events associated with high rates of influenza. Further studies and additional measures are needed to understand additional factors influencing the temporal association between influenza and CV events and to assess new strategies to reduce events.

## Notes

Supplement 1.
Trial Protocol

Supplement 2. **eTable.** Association of Weekly Odds of Primary and Secondary Outcomes With Influenza-Like Illness Activity by Time

Supplement 3.
Data Sharing Statement
