# Supplementary material for: Temporal Association Among Influenza-Like Illness, Cardiovascular Events, and Vaccine Dose in Patients With High-Risk Cardiovascular Disease: Secondary Analysis of a Randomized Clinical Trial
Source: JAMA Netw Open. 2023 Sep 14;6(9):e2331284. doi: 10.1001/jamanetworkopen.2023.31284 (PMC10502520; doi:10.1001/jamanetworkopen.2023.31284)
Supplement: Supplement 2. — eTable. Association of Weekly Odds of Primary and Secondary Outcomes With Influenza-Like Illness Activity by Time [file jamanetwopen-e2331284-s002.pdf]

Supplemental Online Content

Hegde SM, Claggett B, Udell JA, et al. Temporal association among influenza-like illness, cardiovascular events, and vaccine dose in patients with high-risk cardiovascular disease: secondary analysis of a randomized clinical trial. *JAMA Netw Open*. 2023;6(9):e2331284. doi:10.1001/jamanetworkopen.2023.31284

eTable. Association of Weekly Odds of Primary and Secondary Outcomes With Influenza-Like Illness Activity by Time

This supplemental material has been provided by the authors to give readers additional information about their work.

© 2023 Hegde SM, et al. *JAMA Network Open*.

eTable. Association of Weekly Odds of Primary and Secondary Outcomes With Influenza-Like Illness Activity by Time

	Model 1[a] OR (95% CI)	P value	Model 2[b] OR (95% CI)	P value	Model 3[c] OR (95% CI)	P value	Model 4[d] OR (95% CI)	P value
**Lag 0 (0 weeks)**								
Composite Primary Outcome	1.12 (1.06, 1.19)	<0.001	1.12(1.05, 1.19)	0.001	1.11 (1.04, 1.18)	0.001	1.11 (1.04, 1.18)	0.001
Cardiopulmonary Hospitalization	1.12 (1.05, 1.19)	0.001	1.11 (1.04, 1.19)	0.001	1.11 (1.04, 1.18)	0.002	1.11 (1.04, 1.18)	0.002
Death	0.95 (0.84, 1.07)	0.4	0.94 (0.84, 1.07)	0.4	0.94 (0.83, 1.06)	0.3	0.94 (0.83, 1.06)	0.3
Cardiovascular Hospitalization	1.11 (1.03, 1.18)	0.003	1.10 (1.03, 1.18)	0.004	1.09 (1.02, 1.17)	0.008	1.09 (1.02, 1.17)	0.008
Pulmonary Hospitalization	1.09 (0.90, 1.31)	0.4	1.09 (0.90, 1.31)	0.4	1.09 (0.90, 1.31)	0.4	1.09 (0.90, 1.31)	0.4
**Lag 1 (1 week)**								
Composite Primary Outcome	1.15 (1.08, 1.22)	<0.001	1.14 (1.07, 1.22)	<0.001	1.14 (1.07, 1.21)	<0.001	1.14 (1.07, 1.21)	<0.001
Cardiopulmonary Hospitalization	1.14 (1.07, 1.22)	<0.001	1.14 (1.07, 1.21)	<0.001	1.13 (1.06, 1.21)	<0.001	1.13 (1.06, 1.21)	<0.001
Death	1.01 (0.89, 1.14)	0.9	1.00 (0.89, 1.14)	1.0	1.00 (0.88, 1.13)	1.0	1.00 (0.88, 1.13)	1.0
Cardiovascular Hospitalization	1.12 (1.05, 1.20)	<0.001	1.12 (1.05, 1.20)	0.001	1.12 (1.04, 1.19)	0.001	1.12 (1.04, 1.19)	0.001
Pulmonary Hospitalization	1.18 (0.99, 1.39)	0.06	1.18 (0.99, 1.39)	0.06	1.18 (0.99, 1.40)	0.06	1.18 (0.99, 1.40)	0.06
**Lag 2 (2 weeks)**								
Composite Primary Outcome	1.13 (1.06, 1.20)	<0.001	1.13 (1.05, 1.20)	<0.001	1.12 (1.05, 1.20)	<0.001	1.12 (1.05, 1.20)	<0.001
Cardiopulmonary Hospitalization	1.13 (1.06, 1.20)	<0.001	1.12 (1.05, 1.20)	0.001	1.12 (1.05, 1.20)	0.001	1.12 (1.05, 1.20)	0.001
Death	1.01 (0.89, 1.14)	0.9	1.00 (0.88, 1.14)	0.9	1.00 (0.88, 1.14)	0.9	1.00 (0.88, 1.14)	0.9
Cardiovascular Hospitalization	1.12 (1.05, 1.20)	0.001	1.12 (1.04, 1.20)	0.001	1.12 (1.04, 1.19)	0.002	1.12 (1.04, 1.19)	0.002
Pulmonary Hospitalization	1.10 (0.92, 1.32)	0.3	1.10 (0.92, 1.32)	0.3	1.10 (0.92, 1.32)	0.3	1.10 (0.92, 1.32)	0.3

© 2023 Hegde SM, et al. *JAMA Network Open*.

**Lag 3 (3 weeks)**								
Composite Primary Outcome	1.14 (1.08, 1.22)	<0.001	1.14 (1.07, 1.22)	<0.001	1.14 (1.07, 1.22)	<0.001	1.14 (1.07, 1.22)	<0.001
Cardiopulmonary Hospitalization	1.13 (1.06, 1.21)	<0.001	1.12 (1.06, 1.20)	<0.001	1.13 (1.06, 1.21)	<0.001	1.13 (1.06, 1.21)	<0.001
Death	1.08 (0.95, 1.22)	0.3	1.08 (0.95, 1.22)	0.3	1.08 (0.95, 1.22)	0.3	1.08 (0.95, 1.22)	0.3
Cardiovascular Hospitalization	1.13 (1.06, 1.21)	<0.001	1.13 (1.05, 1.21)	0.001	1.13 (1.05, 1.21)	0.001	1.13 (1.05, 1.21)	0.001
Pulmonary Hospitalization	1.07 (0.90, 1.27)	0.5	1/07 (0.90, 1.27)	0.5	1.07 (0.90, 1.28)	0.4	1.07 (0.90, 1.28)	0.4
**Lag 4 (4 weeks)**								
Composite Primary Outcome	1.11 (1.05, 1.19)	0.001	1.11 (1.04, 1.18)	0.001	1.11 (1.05, 1.19)	0.001	1.11 (1.05, 1.19)	0.001
Cardiopulmonary Hospitalization	1.11 (1.04, 1.18)	0.002	1.10 (1.03, 1.18)	0.004	1.11 (1.03, 1.18)	0.003	1.11 (1.03, 1.18)	0.003
Death	1.06 (0.94, 1.20)	0.3	1.06 (0.94, 1.20)	0.3	1.07 (0.94, 1.21)	0.3	1.07 (0.94, 1.21)	0.3
Cardiovascular Hospitalization	1.11 (1.04, 1.19)	0.003	1.11 (1.03, 1.18)	0.004	1.11 (1.03, 1.19)	0.003	1.11 (1.03, 1.19)	0.003
Pulmonary Hospitalization	1.06 (0.90, 1.24)	0.5	1.06 (0.90, 1.24)	0.5	1.06 (0.91, 1.25)	0.5	1.06 (0.91, 1.25)	0.5

[a] Model 1 – adjusted for state
[b] Model 2 – adjusted for Model 1 + age, sex, race, history of myocardial infarction, history of heart failure
[c] Model 3 – adjusted for Model 2 + diabetes mellitus, body mass index >30, renal impairment, current smoker, peripheral arterial disease, ischemic stroke, hypertension, hyperlipidemia, asthma, chronic obstructive pulmonary disease, percutaneous coronary intervention, coronary artery bypass graft, atrial fibrillation, implantable cardiac defibrillator
[d] Model 4 – adjusted for Model 3 + treatment

© 2023 Hegde SM, et al. *JAMA Network Open*.
