# Supplementary material for: Temporal Association Among Influenza-Like Illness, Cardiovascular Events, and Vaccine Dose in Patients With High-Risk Cardiovascular Disease: Secondary Analysis of a Randomized Clinical Trial
Source: JAMA Netw Open. 2023 Sep 14;6(9):e2331284. doi: 10.1001/jamanetworkopen.2023.31284 (PMC10502520; doi:10.1001/jamanetworkopen.2023.31284)
Supplement: Supplement 3. — Data Sharing Statement [file jamanetwopen-e2331284-s003.pdf]

Data Sharing Statement

Hegde. Temporal Association Among Influenza-Like Illness, Cardiovascular Events, and Vaccine Dose in Patients With High-Risk Cardiovascular Disease. *JAMA Netw Open*. Published September 12, 2023. doi:10.1001/jamanetworkopen.2023.31284

Data
Data available: Yes
Data types: Deidentified participant data
How to access data: Data will be available on the NHLBI Biolincc wesite
When available: beginning date: 11/1/2023

Supporting Documents
Document types: None

Additional Information
Who can access the data: Data will be made available to qualified researchers as per NHLBI Guidelines
Types of analyses: Any purpose
Mechanisms of data availability: Through NHLBI BIOLINCC website
Any additional restrictions: None
